# Study on Performance and Aging Mechanism of Rubber-Modified Asphalt Under Variable-Intensity UV Aging

**DOI:** 10.3390/ma18133186

**Published:** 2025-07-05

**Authors:** Qian Liu, Fujin Hou, Dongdong Ge, Songtao Lv, Zihao Ju

**Affiliations:** 1 National Engineering Research Center of Highway Maintenance Technology, Changsha University of Science & Technology, Changsha 410114, China; lq@stu.csust.edu.cn (Q.L.); lst@csust.edu.cn (S.L.);; 2 National Key Laboratory of Green and Long-Life Road Engineering in Extreme Environment (Changsha), Changsha University of Science & Technology, Changsha 410114, China; 3 Shandong Hi-Speed Group, Jinan 250098, China; boyi0706@163.com

**Keywords:** UV aging, rubber-modified asphalt, variable-intensity, rheological properties, aging mechanism

## Abstract

Prolonged ultraviolet (UV) exposure accelerates aging and degradation, while conventional constant-intensity UV simulations do not reflect the variable nature of outdoor radiation. Aging duration and film thickness are both key factors affecting Rubber-Modified Asphalt (RMA), but how their combination influences RMA remains unclear. To address this limitation, this research employed accelerated aging experiments under variable-intensity UV radiation to investigate the performance and aging mechanism of RMA across different aging durations and asphalt film thicknesses. Rheological properties were analyzed through rheological tests, and the UV aging mechanisms of RMA were revealed using FTIR and SEM. The results revealed that crumb rubber improved RMA’s UV aging resistance, including high-temperature performance, fatigue life, and low-temperature cracking resistance. Aging effects were more influenced in RMA with thinner films under prolonged UV exposure. After nine cycles of ultraviolet aging, the rutting resistance, elastic recovery, fatigue life, and low-temperature cracking resistance of RMA with a 1 mm film thickness were 1.33, 1.11, 0.54, and 0.67 times, respectively, those of RMA with a 2 mm film thickness subjected to three UV aging cycles. RMA demonstrated comparable high-temperature performance and elastic recovery under UV aging conditions corresponding to a 1.5 mm film thickness aged for three cycles and a 2.0 mm film thickness aged for six cycles, as well as a 1.0 mm film thickness aged for six cycles and a 1.5 mm film thickness aged for nine cycles. FTIR showed that the increased activity of C=C and C-H under photo-oxidative aging caused a greater impact on the carbonyl groups than the sulfoxide groups. Under high-intensity UV radiation, RMA with thinner films exhibited greater rubber powder detachment, increased surface oxidation, and a substantial widening of cracks. The rubber powder absorbed UV radiation, enhancing the stability of RMA. The maximum crack width of the 1 mm NA was twice that of RMA. These provided insight into the microstructural pattern of cracking resistance degradation caused by aging. This research provides theoretical support for the optimization of the anti-aging performance of RMA.

## 1. Introduction

Compared with unmodified asphalt, the utilization of Rubber-Modified Asphalt (RMA) has attracted considerable attention due to its environmental advantages [1]. Furthermore, it significantly improves pavement performance by enhancing resistance to high-temperature rutting, low-temperature cracking, and fatigue distress [2]. However, prolonged exposure to heat, oxygen, and UV spectral radiation accelerates the aging of RMA, contributing to pavement deterioration [3,4,5]. Although numerous studies have examined the mechanism of oxidative thermal aging, the influence of UV aging has not been sufficiently explored. Consequently, further investigation into the performance evolution and aging mechanism of RMA under UV exposure is essential for promoting its regeneration and reuse.

### 1.1. Heat–Oxidative Aging of Asphalt

The physical behavior and chemical composition of RMA deteriorate under the combined influence of environmental factors and traffic loading, resulting in a decline in pavement performance. Extensive research has examined the oxidative thermal aging behavior of RMA using Rolling Thin Film Oven (RTFO) and Pressure Aging Vessel (PAV) tests, thereby establishing a well-developed theoretical foundation [4,6,7].

Numerous studies have utilized rheological parameters to evaluate the behavior of aged asphalt at high, medium, and low temperatures [8,9,10]. Ji et al. found that after heat-oxidative aging (RTFO and PAV), RMA exhibited improved viscoelasticity, high-temperature behavior, and fatigue durability relative to neat asphalt [5]. Li et al. demonstrated that the high-temperature stability, fatigue durability, and aging resistance of silica-modified RMA increased with higher rubber content [11]. Zhang et al. compared the rheological behavior of RMA and activated rubber-modified asphalts after the PAV test, revealing that activated RMA showed smaller changes in rheological parameters as aging progressed [4]. Chen et al. enhanced the low-temperature performance of aged RMA through surface treatment of various sizes of rubber powder, with the 40-mesh crumb rubber showing the best improvement [12]. Pei et al. indicated that heat-oxidative aging improved the high-temperature behavior of RMA due to changes in chemical composition, physical properties, microstructure, and mechanical properties [13]. Following heat-oxidative aging, RMA’s high-temperature and medium-fatigue behaviors improved, while its low-temperature performance deteriorated.

Heat-oxidative aging involves both the rubber and asphalt binder, as well as their interaction, with the chemical microstructure revealing the underlying aging mechanism [8,9,10]. RTFO and PAV aging are oxidative processes that increase the absorbance of carbonyl and sulfoxide peaks compared to neat asphalt [7,14]. Jiu et al. found that as PAV aging progressed, rubber desulfurization and degradation intensified, leading to the breakage of C=C bonds in the rubber [15]. Several studies have shown that after RTFO and PAV aging, the Large Molecular Size (LMS) of RMA increases, while the Small Molecular Size (SMS) continuously decreases, consistent with the aging behavior of neat asphalt [16,17]. Electron microscopy revealed that the incorporation of rubber reduced surface crack formation in the asphalt binder during heat-oxidative aging [18]. Kim et al. observed a decrease in honeycomb structures in RMA during heat-oxidative aging, likely due to the rubber inhibiting asphalt binder crystallization [19]. After heat-oxidative aging, RMA underwent chemical reactions, leading to increased carbonyl and sulfoxide groups, higher LMS content, and reduced SMS. Additionally, rubber powder mitigated cracks and honeycomb structures.

### 1.2. Ultraviolet Aging of Asphalt

Research on the heat-oxidative aging of RMA is adequate, with extensive studies having evaluated the effects of thermo-oxidative aging on RMA [6,20,21,22]. In contrast, research on ultraviolet aging of RMA is insufficient. The UV aging mechanism of RMA still requires further investigation.

Indoor simulated UV aging conditions involve factors such as light-source wavelength, radiation intensity, asphalt film thickness, aging duration, and temperature. According to the UV radiation spectrum, the typical wavelength range used by researchers is 300–395 nm [23,24]. The asphalt film thickness is generally set between 0.5 and 3.2 mm [25,26]. Considering that the average solar irradiance at the Earth’s surface is approximately 340 W/m^2^, extended aging durations of 50 to 200 h have been employed to simulate the aging of RMA under outdoor UV radiation [27,28]. The temperature range is typically set between 25 °C and 90 °C, considering regional variations [29,30]. Radiation intensity is commonly set at 100–400 W/m^2^ with constant intensity [27,31]. While previous research on UV aging of RMA has utilized constant-intensity radiation, our team introduced a variable-intensity approach to better replicate real-world service conditions [32]. Specifically, using the principle of equal total UV intensity, variable intensity has been applied to simulate the outdoor UV aging process, which was also utilized in this research.

Research on the UV aging performance of RMA has been advanced by several studies. Xu et al. compared RMA and SBS asphalt after 360 h of UV aging, finding that the softening point of RMA decreased by 19 °C, while its penetration and ductility increased slightly by 0.35 mm and 2.4 mm, respectively [29]. Mehdi et al. performed QUV (QUV accelerated weathering tester) aging on asphalt modified with 16.6% and 20% crumb rubber (CR), observing that the rutting parameter increased with aging for 16.6% CR but decreased by 5.3% for 20% CR. The fatigue coefficient was positively correlated with CR content [33]. Jamal and Wu examined the high- and low-temperature performance differences of RMA after heat-oxidative and UV aging. They found that both aging processes increased the complex shear modulus and stiffness modulus, with significant performance changes observed over time [30,34]. Jamal et al. compared RMA with 30-mesh and 50-mesh crumb rubber after UV aging, noting no significant difference in high-temperature rheology, but the 30-mesh RMA exhibited superior elasticity and storage stability [35]. Overall, UV aging improved RMA’s high-temperature performance but reduced its fatigue resistance at intermediate temperatures and its cracking resistance at low temperatures.

Investigating the microscopic reasons for its anti-aging properties and revealing the underlying mechanism hold significant implications for its macroscopic performance. Xu and Gao et al. found that, compared to ultraviolet-aged neat asphalt, RMA exhibited a 5.38% slower decrease in light components and a 4.3% lower LMS conversion rate than SBS asphalt. This was attributed to the protective layer formed by the added ground rubber, which mitigated the UV aging process of RMA [31,36]. Zhang and Jaffer found that, under ultraviolet conditions, rubber powder swelling in RMA reduced polymer crosslinking, causing bond breakage (C-H, C-C, C=C) and an increase in carbonyl groups. This transition from solid/gel to gel-type asphalt worsened low-temperature performance [37,38]. Ultraviolet radiation caused chloroprene rubber and asphalt to react, forming cross-links that roughened and oxidized the rubber surface, reducing low-temperature performance but enhancing high-temperature shear resistance [27].

In summary, research has shown that rubber powder in RMA slows aging and enhances UV resistance. UV aging causes chemical reactions, increasing heavy components, enhancing carbonyl and sulfoxide peaks, and roughening the surface. These microstructural changes improve high-temperature behavior (between 58 °C and 88 °C) but reduce intermediate-temperature (15 °C and 25 °C) and low-temperature (−6 °C, −12 °C, −18 °C and 24 °C) behavior.

RMA is widely used due to its excellent performance. Aging is a key factor affecting its performance. At present, most studies mainly focus on thermo-oxidative aging, while the performance and mechanism of UV aging affecting RMA are still unclear. Existing studies have typically utilized a constant irradiation intensity, neglecting the time-dependent nature of UV radiation in natural environments. Therefore, this study investigates the performance of RMA under variable-intensity UV aging with different film thicknesses and aging cycles.

## 2. Objectives

The effect of intense UV radiation under actual service conditions on the performance of RMA remains unclear. Studying these effects is vital for optimizing the long-term serviceability of RMA mixtures. This study used the real service conditions of the Qinghai-Tibet Plateau as a baseline for simulated aging. An accelerated aging method was employed to replicate the temporal variation in UV radiation and simulate increased UV exposure. Different UV aging cycles were applied to RMA with varying film thicknesses. The rheological properties and microstructural characteristics of RMA under different UV aging conditions were also examined. The aim was to reveal the evolution of rubber properties and the mechanism of UV aging, providing a theoretical foundation for enhancing the long-term performance of RMA.

## 3. Materials and Test Methods

### 3.1. Test Materials

The 70# neat asphalt used in this research was sourced from Maoming City, Guangdong, China. As shown in Table 1, it complied with AASHTO standards [39]. Additionally, 50-mesh (0.3 mm) waste rubber powder particles were obtained from Guangxi Jiaoke Group (Nanning, China), with further details provided in Table 2 [39].

### 3.2. Laboratory Simulation of Outdoor UV Radiation

**Figure 1:**
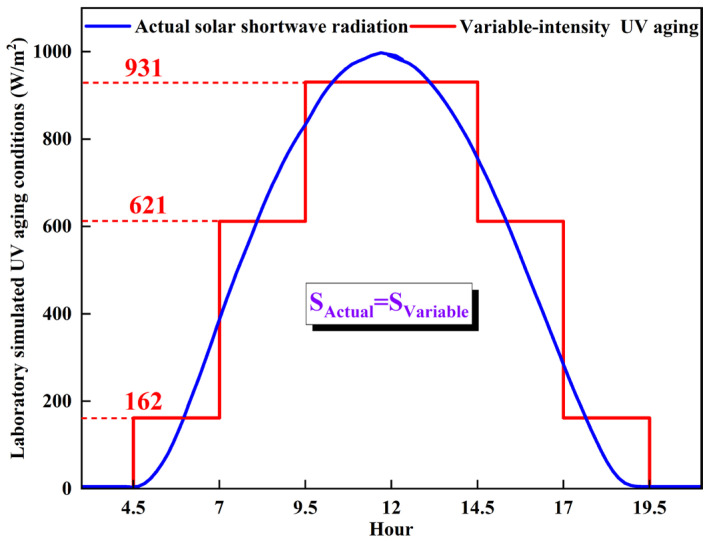
Laboratory UV aging simulation conditions.

As shown in Figure 1, the 15 h UV radiation curve from 4 a.m. to 7 p.m. was divided into six 2.5 h intervals with varying intensities (162, 612, and 931 W/m^2^). This was done to maintain an equivalent cumulative radiation dose (8525 W·h/m^2^) by equalizing the integral areas of the curve and stepped line segments. Each 15 h cycle, equivalent to one month of outdoor radiation in the Qinghai-Tibet region, was used to simulate 2-, 4-, and 6-month UV aging effects on RMA. The ultraviolet radiation wavelength adopted in this paper was 365 nm, serving as the central wavelength of the band. To replicate the summer service temperature, a UV aging temperature of 60 °C was selected [45].

### 3.3. Preparation of UV-Aged RMA

**Figure 2:**
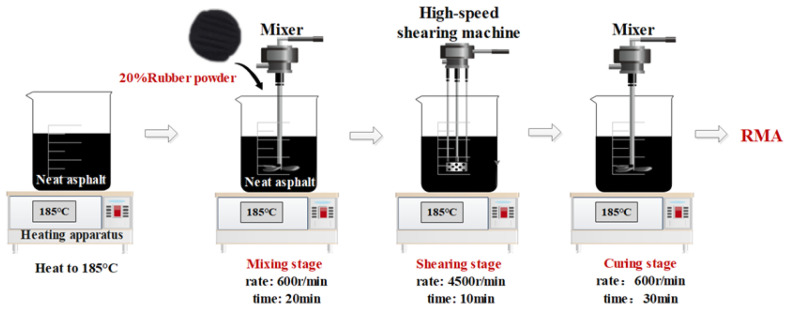
RMA preparation process.

In this study, the rubber particle size was set to 50 mesh (0.3 mm), and the external blending method was employed at 20% of the asphalt mass. Figure 2 provides a detailed illustration of the preparation process.

**Figure 3:**
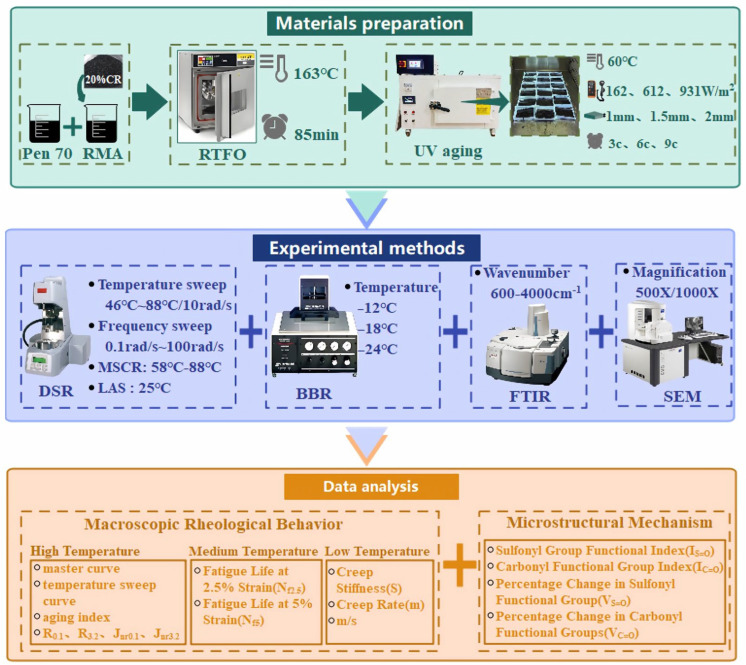
Flowchart of this study.

In accordance with the AASHTO T 240 specification, the RTFO test was performed on RMA to simulate the heat-oxidative aging process during production, transportation, and paving [46]. Short-term aged RMA samples were prepared with asphalt film thicknesses of 1 mm, 1.5 mm, and 2 mm for UV aging simulation. RMA films of different thicknesses were then subjected to indoor ultraviolet aging for 3, 6, and 9 cycles. We surveyed the relevant literature and found that the performance changes of RMA during UV aging are most significant at film thicknesses between 1 mm and 2 mm. Regarding the selection of UV aging cycles for RMA, the differences in property evolution and micro-morphology are most significant in the initial stage of UV aging. To investigate the combined effects of different film thicknesses and aging cycles on RMA, 3, 6, and 9 cycles were chosen in this study [25,26,35,38]. In order to assess the effect of rubber powder on the UV aging of RMA, neat asphalt was prepared under identical conditions. The abbreviations used in this research are presented in Table 3. The research procedure is depicted in Figure 3. For example, UV_N2mm-3c_ represents a 2 mm thick RMA asphalt specimen under three cycles of UV aging.

### 3.4. Test Method

#### 3.4.1. High-Temperature Rheological Tests

High-temperature rheological tests on NA and RMA under various aging conditions were performed using a Dynamic Shear Rheometer (DSR) instrument (Anton Paar MCR 302e dynamic shear rheometer) manufactured by Anton Paar, based in Graz, Austria. Each sample underwent three repeatability tests.

Following the AASHTO T 315 standard, a Temperature Sweep (TS) was conducted within a range of 46 °C to 88 °C at a scanning frequency of 10 rad/s [47].The Aging Index (AI) was employed for quantitative aging analysis.

(1) $$\mathrm{AI}\;=\left(\frac{\mathrm{Rutting}\;\mathrm{factor}\;\mathrm{of}\;\mathrm{aged}\;\mathrm{asphalt}}{\mathrm{Rutting}\;\mathrm{factor}\;\mathrm{of}\;\mathrm{unaged}\;\mathrm{asphalt}}-1\right)\times \;100\%$$

A Frequency Sweep (FS) was performed across frequencies from 0.1 Hz to 10 Hz within the same temperature range. The time–temperature superposition principle was applied to generate complex master curves at various temperatures [48].

According to AASHTO T 350-14, the Multiple Stress Creep Recovery (MSCR) test was performed with a stress of 3.2 kPa and a temperature range of 58 °C to 88 °C [49].

#### 3.4.2. Intermediate-Temperature Fatigue Test

The linear amplitude sweep (LAS) test evaluated the fatigue resistance of neat and rubber-modified asphalts under cyclic loading with increasing amplitude after ultraviolet aging. The DSR instrument was used in accordance with AASHTO TP 101-14 specifications [50]. The test was conducted with a shear strain range of 0% to 30%, a temperature of 25 °C, and a frequency of 10 Hz. Fatigue life at 2.5% and 5% stress levels was determined, with three replicate samples prepared for each asphalt specimen.

#### 3.4.3. Low-Temperature Rheological Test

The low-temperature cracking resistance of RMA after ultraviolet aging was evaluated using the Bending Beam Rheometer (BBR) test, following AASHTO T313 [51]. The BBR instrument (CANNON TE-BBR) was manufactured by Cannon Instrument Company (State College, PA, USA), based in State College, United States. Creep stiffness (S) and the m value, representing the rate of stiffness change, were measured in the conducted test at temperatures of −12 °C, −18 °C, and −24 °C. Three replicates were conducted for each asphalt specimen to ensure the reliability of the results.

#### 3.4.4. Chemical–Microscopic Tests

Fourier Transform Infrared Spectroscopy (FTIR) was employed to analyze the changes in the chemical functional groups of RMA after UV aging. The measurements were conducted using a Thermo Fisher Nicolet iS50 spectrometer (Thermo Fisher Scientific, Dreieich, Germany) with a spectral resolution of 1 cm^−1^. A total of 32 scans were performed over the wavenumber range of 600–4000 cm^−1^. A Smart iTR™ Attenuated Total Reflection (ATR) accessory was adopted to increase the accuracy of test results. Before analyzing the measured spectra, they were vector-normalized by using all data across all spectra and baseline-corrected by the rubber-band method with 64 baseline points, and a plausibility test was carried out. Regarding normalization, this paper follows the procedure described in Hou’s article [52].

To quantitatively evaluate the aging degree, the absorption peaks of sulfoxide (I_S=O_) and carbonyl (I_C=O_) functional groups, along with the Aging Ratio (AR), were calculated as defined by Equations (2)–(4) [53,54].

Scanning Electron Microscopy (SEM) was employed to examine the microstructural characteristics of asphalt specimens. To assess the surface morphology of RMA under severe aging conditions, samples with varying film thicknesses and NA were analyzed after nine cycles of UV aging. A Zeiss EVO10 SEM instrument manufactured by Carl Zeiss AG in Oberkochen, Germany, was used for observation. Prior to imaging at 500× magnification, approximately 1 g of each asphalt sample was gold-coated. Three replicate specimens were prepared for each test condition to ensure the consistency of the results. The crack width calculated by imaging was used to evaluate the cracking degree of the RMA [55].

(2) $$I_{S=O}\;=\frac{A_{1700}}{A_{2800–3000}}$$

(3) $$I_{C=O}\;=\frac{A_{1030}}{A_{2800–3000}}$$

(4) $$V\;=\left(\frac{\mathrm{Functional}\;\mathrm{group}\;\mathrm{index}\;\mathrm{after}\;\mathrm{aging}}{\mathrm{Unaged}\;\mathrm{functional}\;\mathrm{group}\;\mathrm{index}}-1\right)\times 100\%$$

where *A*_1700_, *A*_1030_, and *A*_2800–3000_ refer to the absorbance peak areas at 1700 cm^−1^, 1030 cm^−1^, and areas within the 2800–3000 cm^−1^ range, respectively.

## 4. Results and Discussion

### 4.1. Macroscopic Rheological Behavior

#### 4.1.1. FS Test Results

**Figure 4:**
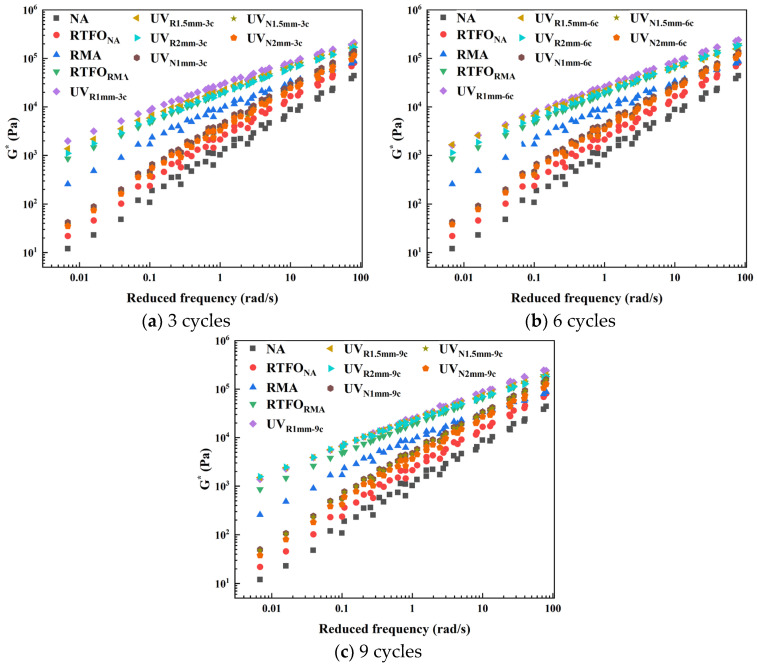
Master curves of asphalt under different UV aging cycles.

Asphalt, being a viscoelastic material, demonstrated temperature-frequency equivalence, where the high-temperature/high-frequency behavior was analogous to the low-temperature/low-frequency behavior. The G*-R master curve was constructed from the FS results, as shown in Figure 4.

The master curve of UV-aged RMA was higher than that of NA, indicating that RMA accumulated residual stress due to a slower dissipation of stress. The incorporation of crumb rubber enhanced the elasticity of RMA, which aligns with the findings of Jamal [35]. The master curve of heat-oxidatively aged RMA was slightly lower than that of UV-aged RMA, suggesting that ultraviolet radiation improved RMA’s resistance to permanent deformation.

Under constant cycles, the order of the RMA master curves was 1 mm > 1.5 mm > 2 mm, indicating that thinner films enhanced the aging effect and improved resistance to high-temperature, low-frequency permanent deformation. For constant film thickness, the order was 9 c > 6 c > 3 c, suggesting that prolonged UV aging increased RMA’s elasticity. RMA samples with thinner films exhibited higher susceptibility to UV penetration, showing a deeper penetration depth and leading to a more pronounced aging effect. Longer aging cycles further improved the deformation resistance of thinner RMA films.

Interestingly, UV-aged RMA showed that the 1.5 mm-3 c and 2 mm-6 c, as well as the 1 mm-6 c and 1.5 mm-9 c curves, coincided, indicating similar resistance to high-temperature deformation under these aging conditions.

#### 4.1.2. TS Test Results

**Figure 5:**
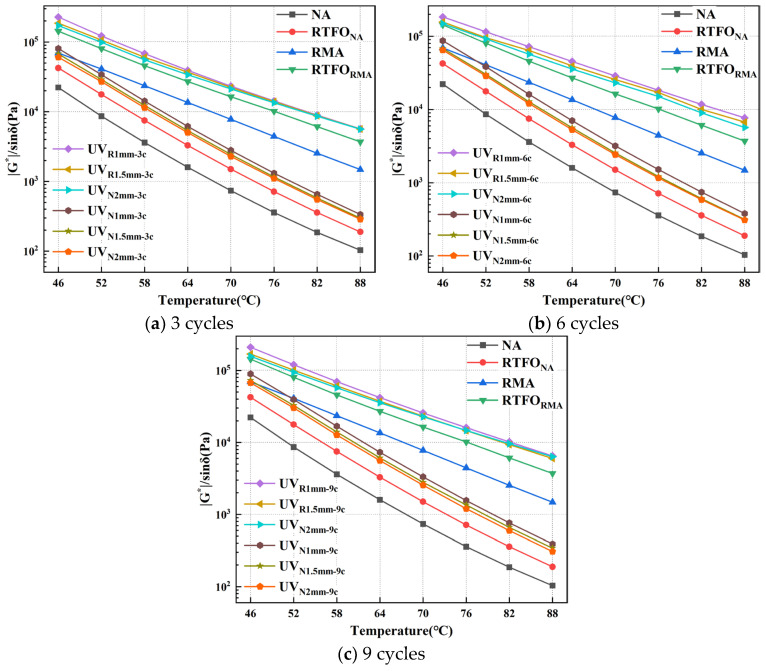
TS results of UV-aged asphalt under different aging cycles.

To evaluate the high-temperature behavior of UV-aged RMA, the rutting parameter–temperature curves for UV-aged RMA and NA at 10 Hz are shown in Figure 5.

Under identical UV aging conditions, RMA consistently exhibited a higher rutting parameter compared to NA. At 64 °C, in comparison to NA, the rutting parameter of RMA increased by 534%, 494.85%, and 522.69% under 1 mm-3 c, 1 mm-6 c, and 1 mm-9 c aging conditions, respectively. The crumb rubber component partially absorbed the lighter fractions of the asphalt, effectively acting as a “sunscreen” under UV radiation, which enhanced the rutting resistance of UV-aged RMA [34].

For a constant asphalt film thickness, the RMA slope order was 9 c > 6 c > 3 c, indicating an increased temperature sensitivity with prolonged UV aging. For constant aging cycles, the slope order was 1 mm > 1.5 mm > 2 mm, with thicker RMA films exhibiting reduced temperature sensitivity. Thinner RMA films became more temperature-sensitive with extended aging, resulting in a more pronounced decrease in G* and increased rutting deformation at elevated temperatures.

**Figure 6:**
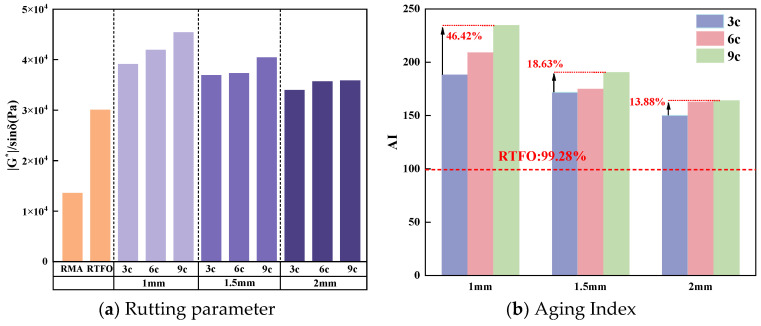
G*/sinδ and AI of RMA at 64 °C.

The rutting parameter of RMA at 64 °C was chosen to investigate its aging behavior under summer conditions, and the aging index was calculated as shown in Figure 6.

As shown in Figure 6a, UV aging increased the G*/sinδ of RMA compared to RTFO, with the values for 1 mm-9 c and 2 mm-3 c being 1.68 and 1.32 times higher, respectively. Photo-oxidative aging improved the high-temperature deformation resistance of RMA by promoting the volatilization of its components. Notably, under the aging conditions of 1.5 mm-3 c and 2 mm-6 c, as well as 1 mm-6 c and 1.5 mm-9 c, the rutting parameters were comparable, which is consistent with the results of the FS tests.

Figure 6b presents the AI calculated from Figure 6a. The AI of RMA under the 1 mm-9 c condition showed the most significant increase. Compared to RTFO-aged asphalt, the rutting index aging index of RMA under the 1 mm-9 c condition increased by 165%. With extended aging cycles, the AI increased, with the largest difference (46.42%) observed at 1 mm thickness and the smallest (13.88%) at 2 mm thickness. Each addition of three aging cycles led to an 11.66%, 5.35%, and 4.59% increase in the AI for 1 mm, 1.5 mm, and 2 mm thicknesses, respectively. For constant aging cycles, the AI increased with asphalt film thickness. Each 0.5 mm increase in thickness decreased the AI by 11.96%, 13.45%, and 19.63% for the 3 c, 6 c, and 9 c cycles, respectively. RMA with thinner films experienced more severe aging effects as UV cycles increased, with the rutting factor of 1 mm-9 c being 1.33 times that of 2 mm-3 c.

The addition of rubber enhanced the UV aging resistance of RMA. Prolonged UV aging led to more pronounced high-temperature aging effects in RMA with thinner films. At elevated temperatures, RMA’s rutting resistance was comparable under the 1.5 mm-3 c, 2 mm-6 c, 1 mm-6 c, and 1.5 mm-9 c aging conditions.

#### 4.1.3. MSCR Test Results

**Figure 7:**
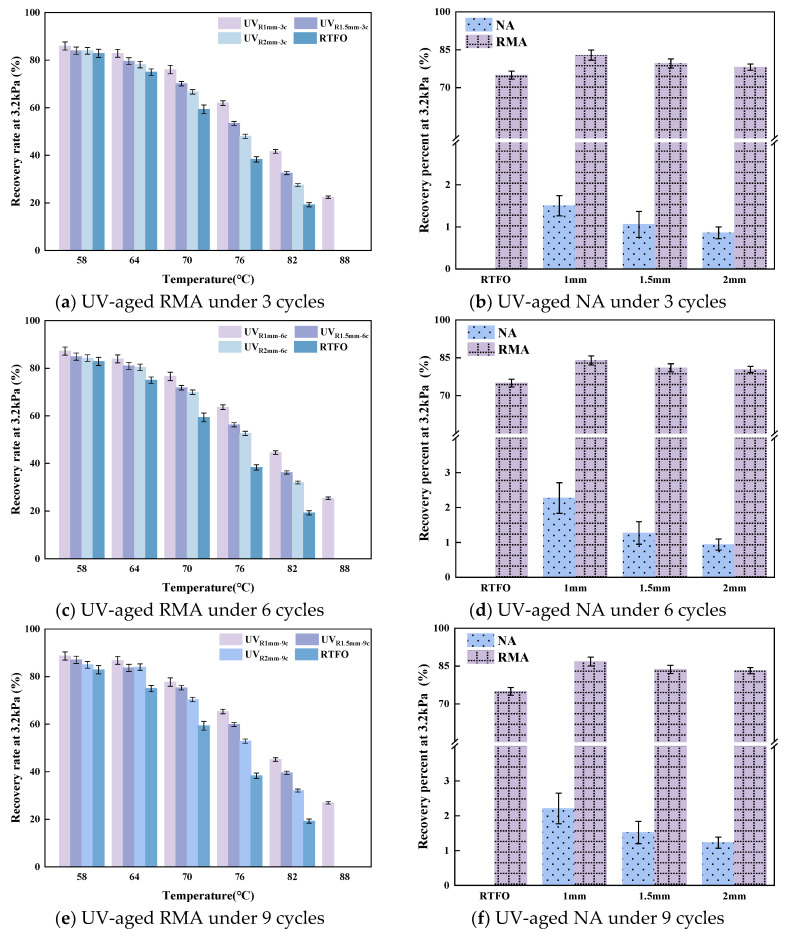
The R value of asphalt under 3.2 kPa for different aging cycles.

The average recovery rate (R) and non-recoverable creep compliance (J_nr_) were used to assess RMA’s deformation and recovery behavior under high-temperature loading. Figure 7a,c,e present the R values of UV-aged RMA under 58–88 °C and 3.2 kPa loading, while Figure 7b,d,f show the R values of UV-aged neat asphalt (NA) and RMA at 64 °C.

**Figure 8:**
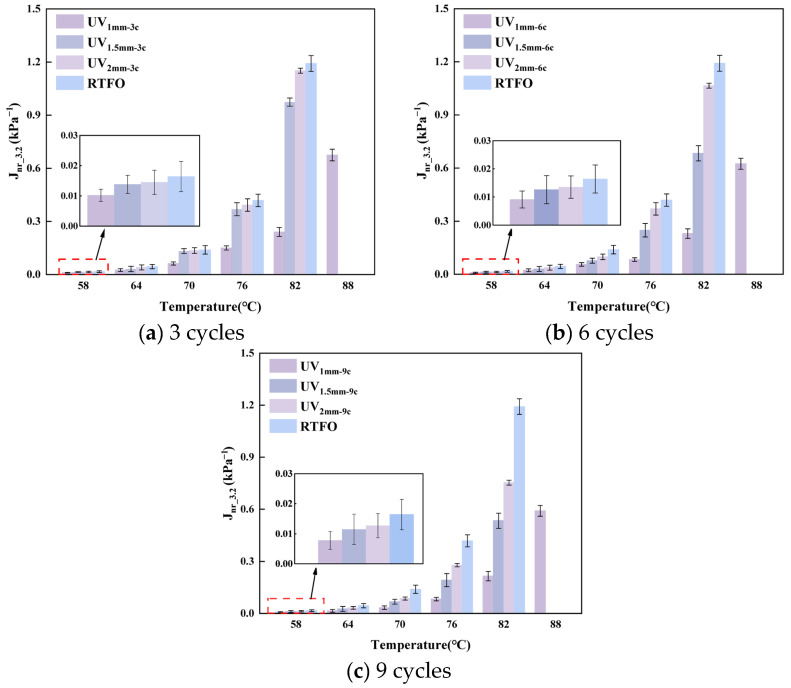
J_nr_ of RMA under different UV aging cycles.

As shown in Figure 7a,c,e, the R value of UV-aged RMA decreased with increasing temperature, with only the 1 mm film maintaining elasticity at 88 °C. Photo-oxidative aging resulted in a higher R value for RMA compared to heat-oxidative aging, with the gap widening at elevated temperatures. This difference was likely due to the volatilization of lighter components, which enhanced elasticity. As shown in Figure 7b,d,f, at 64 °C, the R value of both UV-aged RMA and NA decreased as film thickness increased but increased with longer aging cycles. Under the 1 mm-9 c aging condition, RMA’s R value increased by 84.59% compared to NA, indicating that crumb rubber improved elasticity behavior. Regarding Figure 8, the differences in MSCR test results for different film thicknesses under various cycles are relatively small. The possible reasons for the small differences include that the viscoelastic states (such as glassy state and rubbery state) of the material change significantly at different temperatures, which may exceed the influence of film thickness and aging cycles [26,56]. Cross-linking reactions (such as the formation of carbonyl groups and sulfoxide groups) increase the rigidity of molecular chains, which may improve the elastic recovery ability [32]. UV aging leads to reductions in light components, making the material harder and more brittle [36]. However, under different film thicknesses, the loss rate of light components may be compensated for by intermolecular recombination, maintaining the relative stability of the viscoelastic structure, thereby making the difference in recovery rate not significant. Notably, RMA’s R value was 16% higher than that of RTFO-aged asphalt.

At 64 °C, the R value increased after three aging cycles by 3.18%, 2.54%, and 2.35% for film thicknesses of 1 mm, 1.5 mm, and 2 mm, respectively. For a constant aging cycle, the R value decreased as the film thickness decreased. Specifically, each 0.5 mm increase in thickness resulted in reductions of 2.16%, 2.19%, and 3.00% in R under 3 c, 6 c, and 9 c cycles, respectively. As the UV aging duration increased, the elasticity of thinner RMA films improved, leading to a decrease in non-recoverable deformation. The reason was that under prolonged UV radiation, the RMA with thinner films experienced an increase in heavy-component content, which, in turn, enhanced their elastic properties. Specifically, the R value of the 1 mm-9 c sample was 1.11 times that of the 2 mm-3 c sample. With extended aging cycles, RMA with thinner films demonstrated superior elastic recovery, maintaining strong performance under high-stress and high-temperature conditions. Notably, the R values under 1.5 mm-3 c and 2 mm-6 c, as well as 1 mm-6 c and 1.5 mm-9 c aging conditions, were comparable.

J_nr_ reflects the irreversible deformation capacity of asphalt under prolonged loading, with lower values indicating better resistance. Figure 8 illustrates the non-recoverable creep compliance of UV-aged RMA at 3.2 kPa, while Table 4 presents the Jnr values of both RMA and NA at 64 °C.

In the 58–88 °C temperature range, J_nr_ increased with rising temperature, while UV-aged RMA’s J_nr_ remained below 0.003 Pa^−1^. For constant aging cycles, the J_nr_ values followed the order of 2 mm > 1.5 mm > 1 mm, and for constant film thickness, the order was 3 c > 6 c > 9 c. High shear forces concentrated at the interface between the surface and the interior of RMA lead to a decrease in interface shear strength. This results in irreversible flow of the internal asphalt under shear stress, causing greater non-recoverable deformation.

At 64 °C, the J_nr_ value of RMA decreased with increasing UV aging cycles at a constant film thickness. Specifically, for film thicknesses of 1 mm, 1.5 mm, and 2 mm, every additional three aging cycles reduced the J_nr_ by 30.98%, 18.76%, and 12.41%, respectively. Conversely, under constant aging cycles, J_nr_ increased with film thickness. Each 0.5 mm increment resulted in increases of 26.8%, 28.31%, and 46.51% under the 3 c, 6 c, and 9 c aging conditions, respectively. Prolonged UV exposure reduced the irreversible deformation capacity of thinner RMA films, with the J_nr_ of the 1 mm-9 c condition being only 0.37 times that of the 2 mm-3 c condition. Notably, J_nr_ values under the 1.5 mm-3 c and 2 mm-6 c conditions, as well as the 1 mm-6 c and 1.5 mm-9 c conditions, were comparable.

Crumb rubber greatly enhanced the elasticity of RMA, which remained stable after UV aging. Under high-temperature, high-stress conditions, RMA with thinner films exhibited enhanced elastic deformation and increased resistance to non-recoverable deformation with prolonged UV exposure. The elastic deformation and recovery were comparable under both aging conditions, consistent with the rutting resistance behavior.

#### 4.1.4. LAS Test Results

**Figure 9:**
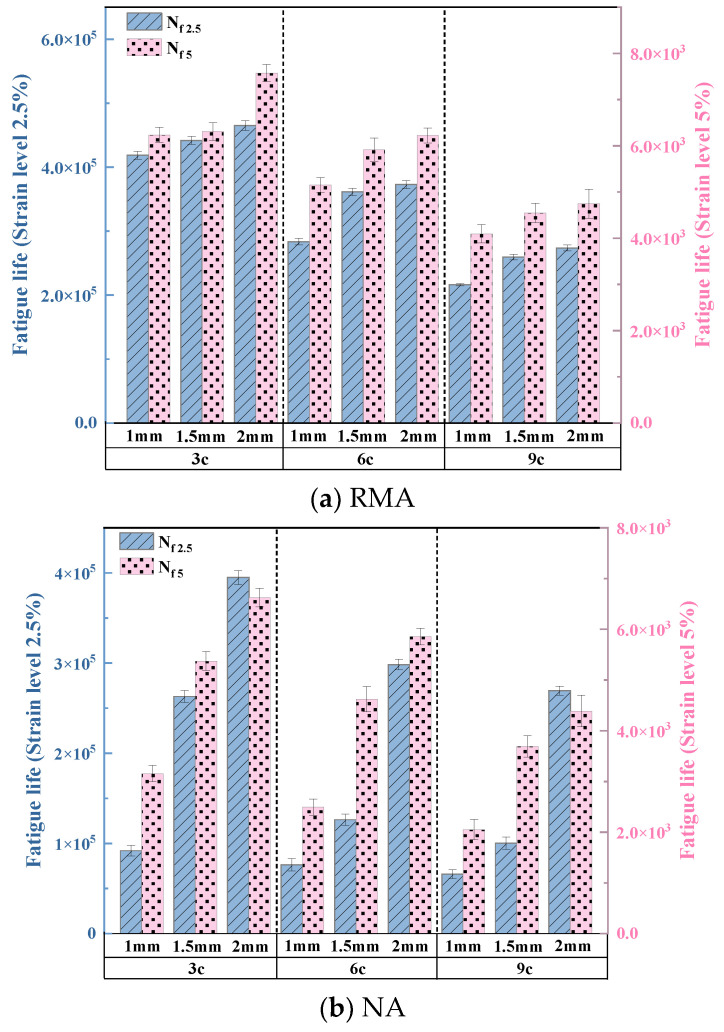
Fatigue life of UV-aged asphalt.

The influence of UV aging on the mid-temperature fatigue behavior of RMA was investigated. The average fatigue life of RMA and NA under 2% and 5% strain was calculated as shown in Figure 9.

As presented in Figure 9, the fatigue life of RMA exceeded that of NA, exhibiting a more gradual variation. Under varying stresses, the fatigue life of 1 mm-9 c RMA was 3.28 times (2.5% strain) and 1.99 times greater (5% strain) than that of NA. This improvement was ascribed to the UV-blocking effect of carbon black in the crumb rubber and the enhanced fatigue resistance from the interaction between crumb rubber and asphalt [34].

Figure 9b illustrates that with extended aging cycles, the fatigue life of RMA decreased for all film thicknesses. Specifically, for 1 mm, 1.5 mm, and 2 mm films, each additional three UV aging cycles reduced N_f2.5_ by 39.36%, 30.78%, and 30.52%, respectively, and N_f5_ by 26.45%, 23.43%, and 18.37%, respectively. For constant aging cycles, thinner films exhibited shorter fatigue lives. Under the 3 c, 6 c, and 9 c conditions, each 0.5 mm increase in film thickness led to increases in N_f2.5_ of 5.41%, 12.69%, and 15.33%, respectively, and in N_f5_ of 7.7%, 10.03%, and 10.63%, respectively. Prolonged UV aging reduced the fatigue resistance of RMA with thinner films. The fatigue life of 1 mm-9 c RMA was 0.47 and 0.54 times that of 2 mm-3 c RMA under 2.5% and 5% loads, respectively.

After UV aging, RMA demonstrated significantly higher fatigue life and superior fatigue resistance compared to NA. However, with extended UV exposure, the fatigue resistance of thicker RMA films deteriorated.

#### 4.1.5. BBR Test Results

**Figure 10:**
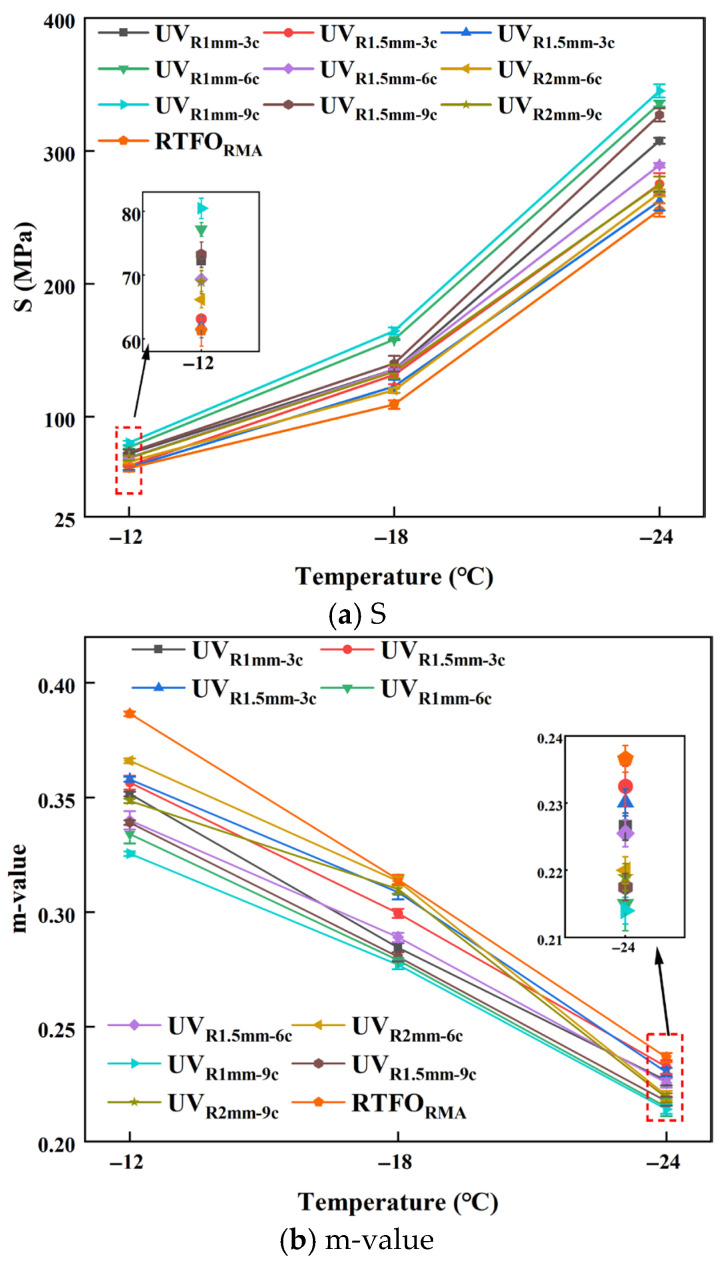
BBR test results of UV-aged RMA.

The cracking resistance of UV-aged RMA at low temperatures was examined at −12 °C, −18 °C, and −24 °C, as illustrated in Figure 10 and detailed in Table 5.

As illustrated in Figure 10 and Table 5, the creep stiffness (S) value of NA was consistently higher than that of RMA. Under −18 °C, the S value of NA increased to three times that of RMA at 1 mm-9 c, while the m value increased to 0.9 times that of RMA. This indicates that crumb rubber improved the low-temperature cracking resistance of RMA under UV aging, conferring UV aging resistance. Under the 1 mm-9 c aging condition, RMA exhibited the highest S value and the lowest m value. The S value increased by 261.9% compared to RTFO asphalt, while the m value decreased by 12.32%, similar to the conclusions of Wu’s study [30].

As presented in Figure 10a, for a constant film thickness, the S value increased with prolonged aging cycles. At −18 °C, the S value increased with aging cycles. For 1 mm, 1.5 mm, and 2 mm film thicknesses, each additional three UV aging cycles raised the S value by 10.36%, 4.48%, and 3.18%, respectively. For a constant aging cycle, increasing the film thickness resulted in a reduction in the S value. Specifically, under the 3 c, 6 c, and 9 c aging conditions, each 0.5 mm increase in film thickness led to a decrease in the S value of 5.06%, 10.99%, and 14.36%, respectively. Prolonged UV aging cycles reduced the low-temperature cracking resistance of thinner RMA films. Under 1 mm-9 c aging condition, the S value of RMA increased by 33.96% compared to 2 mm-3 c aging.

**Figure 11:**
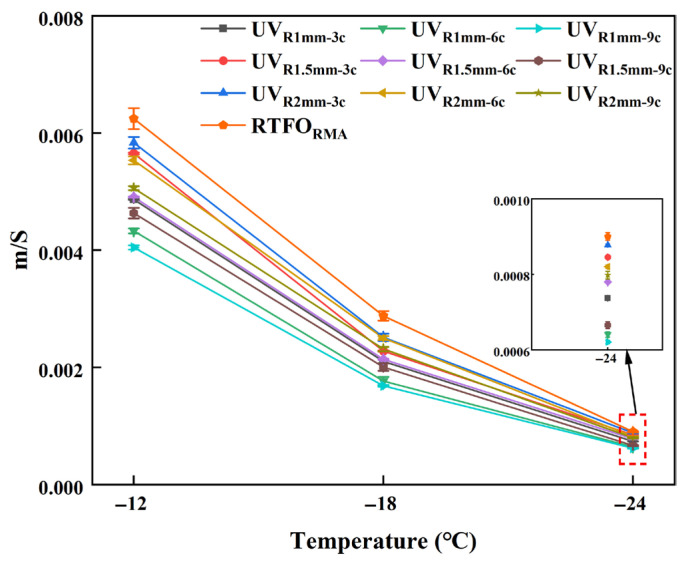
m/S results of UV-aged RMA.

Figure 10a demonstrates that the S value exhibited a positive correlation with temperature. However, the trend of the m value for UV-aged RMA varied at different temperatures. Therefore, Liu proposed using the m/S ratio to evaluate the low-temperature performance of asphalt [57], as shown in Figure 11.

As shown in Figure 11, the m/S ratio decreased with temperature, indicating a deterioration in the low-temperature cracking resistance of UV-aged RMA. The highest m/S value was observed for RTFO, while the lowest was for the 1 mm-9 c aging condition of RMA. At three temperatures, the m/S value decreased by 54.32%, 70.87%, and 45.13%, with the most significant reduction occurring at −18 °C. This decline can be attributed to molecular changes in the crumb rubber induced by UV aging and oxidation at the crumb rubber–asphalt interface, which adversely affected the low-temperature mechanical properties [3].

With constant film thickness, the m/S ratio increased with prolonged aging cycles. At −18 °C, each additional three UV aging cycles increased the m/S ratio by 11.89%, 6.62%, and 4.27% for asphalt films with thicknesses of 1 mm, 1.5 mm, and 2 mm, respectively. For a constant aging cycle, the m/S ratio decreased with increasing film thickness. Specifically, under 3 c, 6 c, and 9 c cycles, each 0.5 mm increase in thickness led to a reduction in the m/S ratio of 9.41%, 17.23%, and 19.05%, respectively, which was consistent with the results of the creep stiffness analysis. Under 1 mm-9 c UV aging conditions, the m/S value of RMA was 0.67 times that of the 2 mm-3 c RMA. Longer UV aging cycles in RMA with thinner films led to poor adhesion and interface defects. These issues caused an uneven stress distribution and microcrack formation, which impaired low-temperature performance. Additionally, at −12 °C and −24 °C, the m/S value of 1.5 mm-3 c was slightly higher than that of 2 mm-6 c, with the observed differences attributed to the synergistic effects of film thickness and aging cycles.

The incorporation of crumb rubber improved the low-temperature cracking resistance of UV-aged RMA. With extended UV aging cycles, RMA with thinner films exhibited increased brittleness and hardness, with 1 mm-9 c aging resulting in a 33% reduction in low-temperature performance compared to 2 mm-3 c aging.

### 4.2. Microstructural Mechanism

#### 4.2.1. FTIR

**Figure 12:**
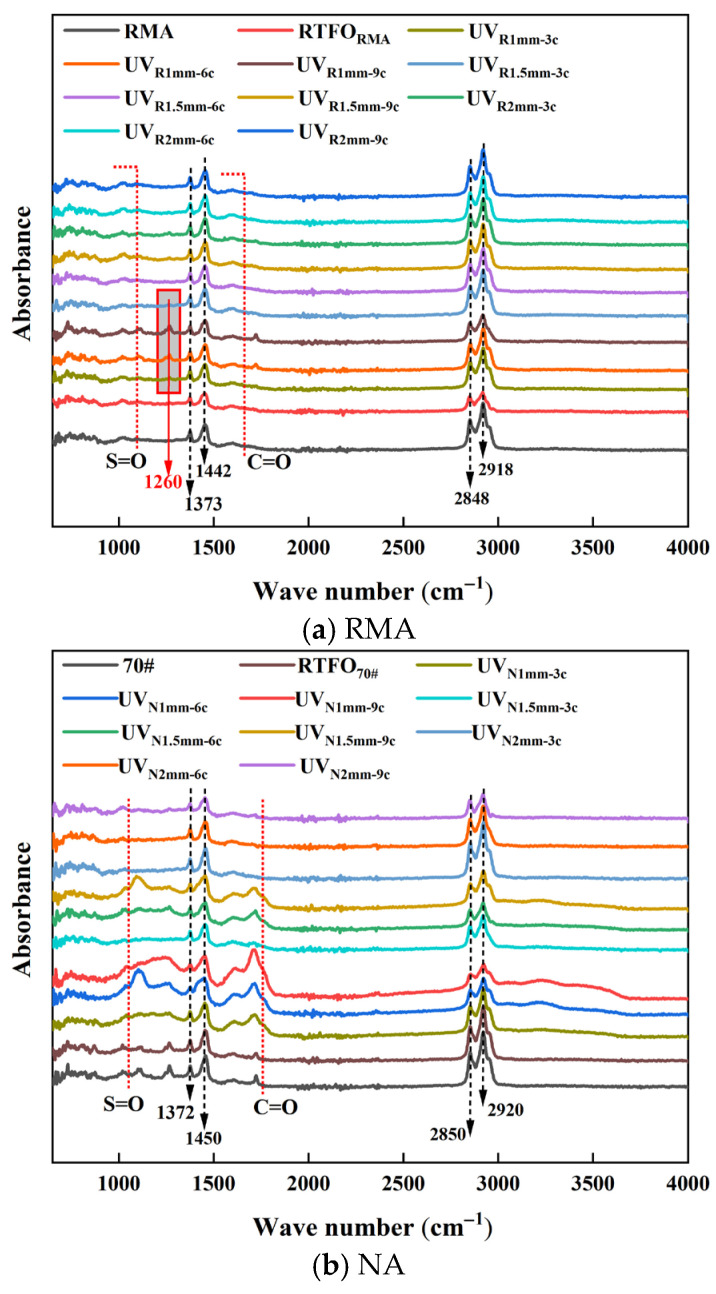
Infrared absorption peaks of asphalt under UV aging.

Infrared spectroscopy was employed to analyze the absorption peaks of asphalt and examine the variations in peak area under different UV aging conditions and film thicknesses, as shown in Figure 12.

Two distinct absorption peaks of asphalt were observed near 2920 cm^−1^ and 2850 cm^−1^, corresponding to the C-H stretching vibration of unsaturated carbon and the asymmetric C-H stretching vibration in the methylene group [58,59]. The peak near 1700 cm^−1^ was attributed to the C=O stretching vibration, while those at 1442 cm^−1^ and 1373 cm^−1^ corresponded to the scissoring vibration of -CH_2_- and the symmetric deformation of -CH_3_, respectively [15,60]. After aging, a notable sulfone (S=O) stretching vibration appeared near 1030 cm^−1^ [32].

Both carbonyl and sulfone groups appeared in RMA after thermal oxidation aging. However, after UV aging, the peak areas of these groups in RMA slightly increased, indicating oxidation caused by UV exposure. After UV aging, a new peak appeared at 1260 cm^−1^. Additionally, the vibration of carbonyl groups after UV aging may be associated with the formation of ether (C-O single bonds) [61]. Compared to NA, significant changes in carbonyl and sulfone groups were observed after short-term and UV aging, while RMA exhibited only minor changes. This was attributed to crumb rubber, which provided both a physical barrier and a chemical system that absorbed UV energy, thereby reducing singlet oxygen and free radical formation [62].

**Figure 13:**
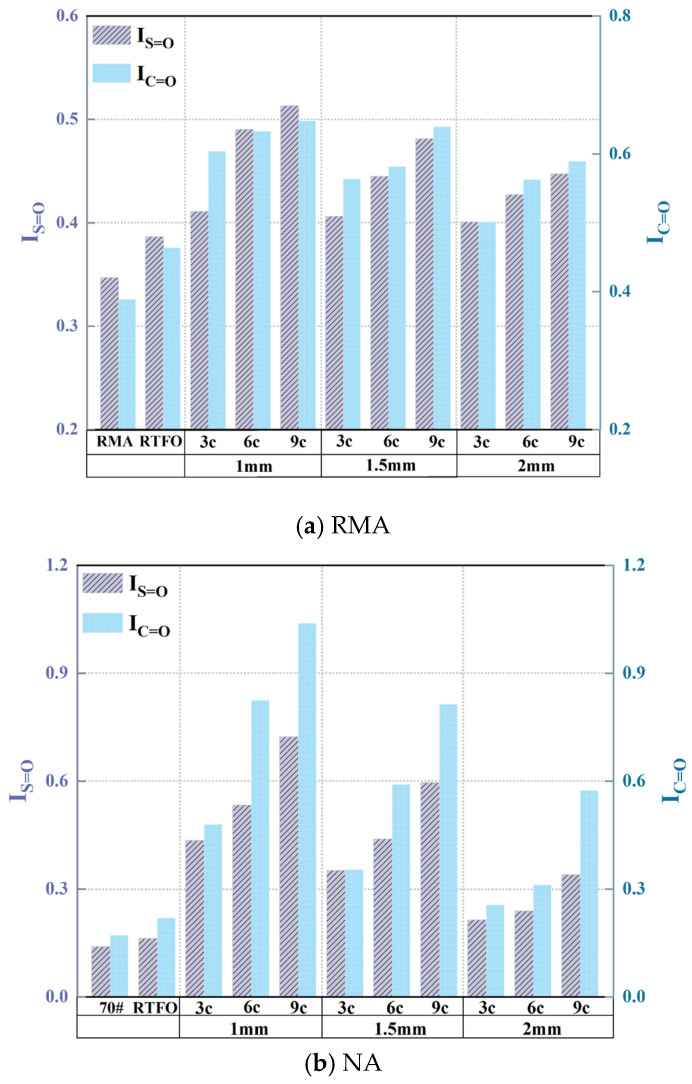
Functional group indices of UV-aged asphalt.

**Figure 14:**
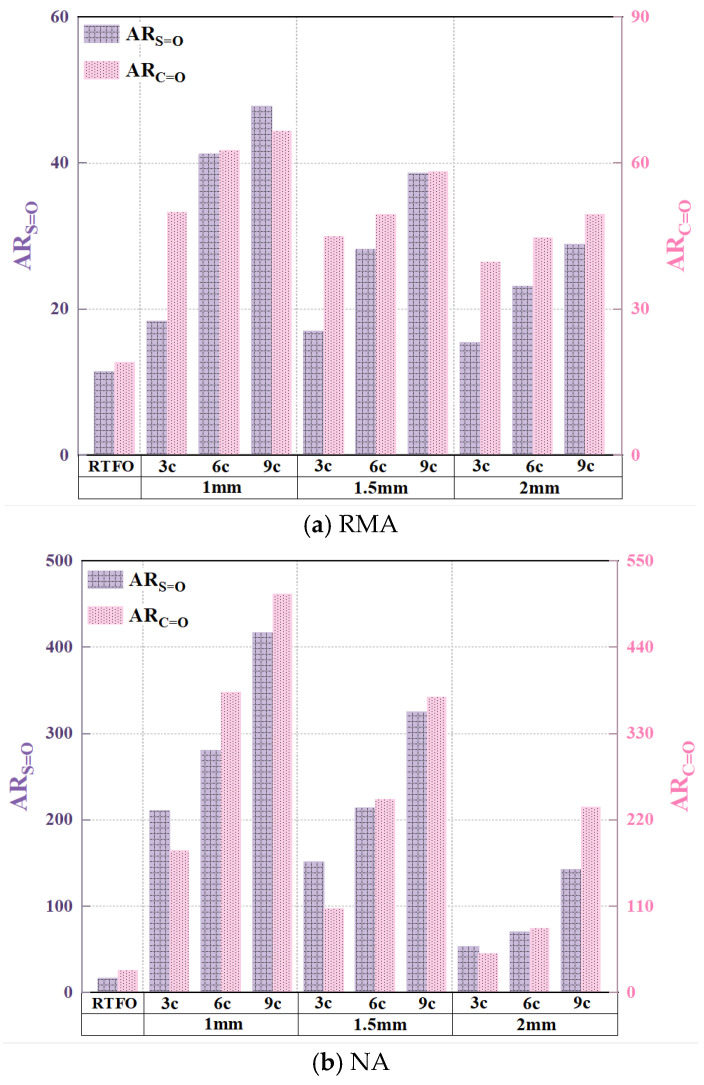
Aging rate of asphalt functional groups.

To further explore the impact of aging conditions on RMA, the sulfone and carbonyl functional group indices, as well as the aging rate, were calculated as shown in Figure 13 and Figure 14.

Figure 13 and Figure 14 showed that UV aging increased the functional group indices of both RMA and NA, with NA exhibiting more significant fluctuations. Under the 1 mm-9 c aging condition, the sulfoxide and carbonyl indices of NA were 29.0% and 37.64% higher than those of RMA, respectively. This phenomenon was attributed to the antioxidant effect of carbon black in crumb rubber. RMA displayed the highest functional group aging rate under the 1 mm-9 c UV condition, with the sulfonyl and carbonyl indices increasing by 318.9% and 247.40%, respectively, compared to RTFO.

As observed in Figure 14, the carbonyl aging rate was higher than that of the sulfoxide group, with RMA’s carbonyl group being more significantly affected by UV aging. Carbon–carbon double bonds and carbon–hydrogen bonds were more susceptible to photo-oxidative aging, whereas sulfur oxidation occurred at a slower rate. As the UV aging cycles increased, the aging rate also increased for all film thicknesses. The increase in AR_C=O_ value per three cycles was 15.9%, 14.0%, and 11.6% for film thicknesses of 1 mm, 1.5 mm, and 2 mm, respectively. The aging rate decreased as the film thickness increased. For 3-cycle, 6-cycle, and 9-cycle tests, the decrease in AR_C=O_ value per 0.5 mm increase in thickness was 17.7%, 16.93%, and 12.1%, respectively, with sulfoxide exhibiting a similar trend. Under the 1 mm-9 c aging condition, RMA’s functional aging rates were 47.9% (S=O) and 66.6% (C=O). The carbonyl group was 18.7% higher than the sulfoxide group.

Under UV aging, degradation of crumb rubber in RMA led to the formation of new substances, including a functional group at 1260 cm^−1^ for the 1 mm film thickness. As UV aging progressed, the film thickness decreased, and the activity of functional groups increased. The carbonyl group was more susceptible to photo-oxidative aging.

#### 4.2.2. SEM

**Figure 15:**
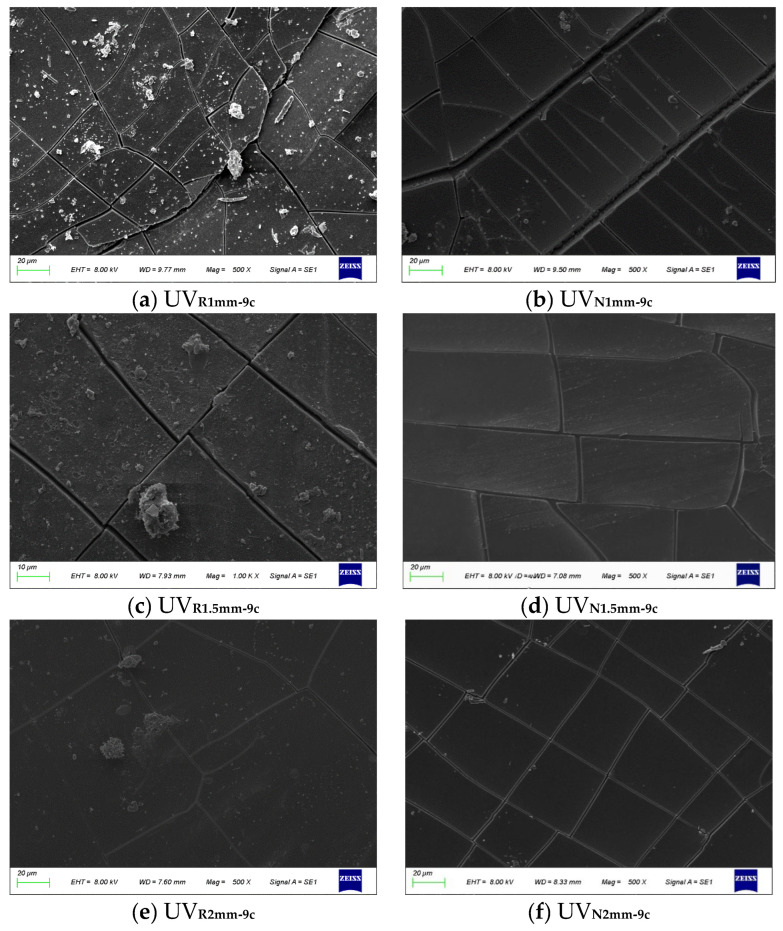
SEM results of asphalt.

SEM was employed to analyze the distribution of crumb rubber particles and the structural damage in crumb RMA subjected to prolonged variable UV radiation, as illustrated in Figure 15 and Table 6.

RMA exhibited markedly different morphological characteristics compared to NA. Under the 1 mm-9 c aging condition (Figure 15a,b), the maximum crack width of NA was twice that of RMA. NA displayed a regular crack network with deeper transverse cracks, revealing its internal structure. In contrast, RMA became brittle, fragmented into irregular pieces, and exhibited shallow cracks, with its internal structure remaining intact. Under 1.5 mm and 2 mm conditions (Figure 15c–f), the crack width of NA was 1.66 and 1.5 times greater than that of RMA, respectively, with NA’s surface morphology being less stable. The incorporation of crumb rubber in long-term, high-intensity outdoor UV radiation environments contributed to the maintenance of the surface stability of RMA.

Figure 15a displays dense, irregular radial cracks with a maximum width of 3.64 μm, indicating severe oxidation due to extensive UV penetration. The surface brittleness resulted in delamination at the RMA interface. In Figure 15c, a more uniform crack network can be observed, with a maximum width of 2.73 μm, and some poorly defined crumb rubber particles visible at the interface. Figure 15e shows fewer, larger cracks, each under 1 μm, with intact crumb rubber particles and a dense oxidized film on the surface. Under prolonged UV exposure, the maximum crack width decreased by 33.35% and 164.4% for each 0.5 mm increase in thickness within the 1–1.5 mm and 1.5–2 mm ranges, respectively. Thicker RMA films exhibited a more uniform crumb rubber distribution, which absorbed UV radiation and slowed oxidation, while films with more dispersed crumb rubber oxidized more rapidly.

Crumb rubber particles absorbed UV radiation, mitigating surface damage in RMA. Thinner RMA films exhibited increased crack growth and reduced stability under prolonged UV aging. Controlling asphalt film thickness is crucial for optimizing both durability and cost-effectiveness.

## 5. Conclusions

This study considered the effects of asphalt film thickness and aging cycles on RMA. Variable-intensity UV radiation was used to simulate outdoor UV aging of RMA and NA. Rheological properties were analyzed through rheological tests, and microscopic techniques were used to reveal the UV aging mechanism of RMA. The research findings are summarized as follows:

(1) The addition of rubber powder significantly improved the UV aging resistance of RMA. The RMA with 1 mm film thickness after nine aging cycles exhibited the most pronounced changes. Under 1 mm-9 c aging conditions, RMA outperformed NA in rutting resistance, elastic recovery, fatigue life, and low-temperature cracking resistance by 5, 1.83, 3.29, and 3 times, respectively.
(2) Aging effects were more pronounced in RMA with thinner films under prolonged UV exposure. Under 1 mm-9 c UV conditions, the rutting resistance, elastic recovery, fatigue life, and low-temperature cracking resistance of RMA were 1.33, 1.11, 0.54, and 0.67 times those of RMA with 2 mm-3 c, respectively. Under different ultraviolet aging conditions, similar effects were achieved. High-temperature performance and elastic recovery ability were comparable under 1.5 mm-3 c and 2 mm-6 c, as well as 1 mm-6 c and 1.5 mm-9 c conditions.
(3) The increased activity of C=C and C-H under photo-oxidative aging caused a greater impact on the carbonyl groups than the sulfoxide groups. For the RMA with a 1 mm film thickness after nine cycles of UV aging, the aging rate of carbonyl groups was 18.7% higher than that of sulfoxide groups. The presence of carbon black in the rubber mitigated the reactivity of chemical functional groups in RMA under UV radiation.
(4) SEM analysis showed that under prolonged UV radiation, RMA with thinner films exhibited increased rubber powder detachment, deeper oxidation, wider cracks, and irregular surface cracks. Rubber powder absorbed UV radiation, maintaining the stability of RMA. The maximum crack width of the 1 mm NA film was twice that of RMA. This revealed the pattern of crack resistance degradation due to aging from a microstructural perspective.

## 6. Future Research Work

This study focused solely on investigating the UV aging performance and mechanism of RMA. Future research will examine the UV aging performance of rubber-modified asphalt mixtures in road applications, supplementing the current understanding of UV aging in RMA. Furthermore, research will establish a correlation between indoor simulated aging and outdoor mixture aging extraction.

## Figures and Tables

**Table 1:** The performance parameters of neat asphalt.

Property	Measured Value	Reference Standard	Specification
Penetration (0.1 mm)	71	AASHTO T 49 [40]	60–80
Ductility (cm)	108.1	AASHTO T 51 [41]	≥100
Softening point (°C)	51.2	AASHTO T 53 [42]	≥46
Density (g/cm^3^)	1.028	AASHTO T 228 [43]	-
Flash point (°C)	302	AASHTO T 48 [44]	≥260

**Table 2:** The properties of rubber powder.

Property	Test Result
Density (g/cm^3^)	1.18
Carbon black content (mass ratio, %)	29
Ash content (mass ratio, %)	3
Acetone extract (mass ratio, %)	7
Rubber hydrocarbon content (mass ratio, %)	56

**Table 3:** Abbreviations of asphalt samples.

Asphalt Type	Asphalt Film Thickness	Aging Cycle	Abbreviation
Neat asphalt	/	/	NA
Rubber-modified asphalt	/	/	RMA
RTFO-aged neat asphalt	/	/	RTFO_NA_
RTFO-aged rubber-modified asphalt	/	/	RTFO_RMA_
Ultraviolet-aged neat asphalt	x = 1 mm/1.5 mm/2 mm	y = 3 c/6 c/9 c	UV_Nxmm-yc_
Ultraviolet-aged rubber-modified asphalt	x = 1 mm/1.5 mm/2 mm	y = 3 c/6 c/9 c	UV_Rxmm-yc_

**Table 4:** J_nr_ of RMA and NA.

Type	J_nr_ at 3.2 kPa (kPa^−1^)	
	Neat Asphalt	Rubber-Modified Asphalt
RTFO	3.2408	0.0448
1 mm-3 c	1.6292	0.0253
1.5 mm-3 c	1.8692	0.0342
2 mm-3 c	1.9529	0.0405
1 mm-6 c	1.5466	0.0228
1.5 mm-6 c	1.7223	0.0302
2 mm-6 c	1.8933	0.0375

**Table 5:** BBR test results of UV-aged NA.

Asphalt Type	−12 °C		−18 °C		−24 °C	
	S	m	S	m	S	m
NA_RTFO_	128.7	0.3962	371.5	0.3165	795.5	0.218
UV_N1mm-3c_	143.4	0.2811	380.6	0.3075	808.6	0.206
UV_N1.5mm-3c_	138.6	0.2839	376.1	0.3186	803.2	0.211
UV_N2mm-3c_	135	0.2847	373.9	0.3203	798.5	0.215
UV_N1mm-6c_	155.3	0.2783	388.5	0.2966	815.3	0.198
UV_N1.5mm-6c_	149.6	0.2795	383.3	0.3024	811.9	0.203
UV_N2mm-6c_	136.2	0.2892	374.6	0.3112	797.1	0.213
UV_N1mm-9c_	164.7	0.2774	395.2	0.2755	823.2	0.177
UV_N1.5mm-9c_	160.1	0.2766	389.6	0.2936	816.6	0.195
UV_N2mm-9c_	155.2	0.2799	384.5	0.2977	809.3	0.199

(Note: The values in this table represent the average values).

**Table 6:** Maximum crack width of asphalt.

Asphalt Types		Maximum Width of Cracks (μm)
1 mm-9 c	NA	10.91
	RMA	3.64
1.5 mm-9 c	NA	7.27
	RMA	2.73
2 mm-9 c	NA	1.82
	RMA	0.73

(Note: The maximum crack width is the average of five scanned images from the same specimen).

## Data Availability

The original contributions presented in this study are included in the article. Further inquiries can be directed to the corresponding author.

## References

[1] Ge D., Jiang X., Lyu S., Zhang H., Song X. (2024). Research progress of rubber-modified asphalt by wet process. J. China Foreign Highw..

[2] Jiao B., Gong M. (2024). Multiscale enhancement mechanism of low-temperature performance for degraded recycled waste rubber asphalt binders: MD simulation and microscopic investigation. J. Clean. Prod..

[3] Mohamed A.S., Cao Z., Xu X., Xiao F., Abdel-Wahed T. (2022). Bonding, rheological, and physiochemical characteristics of reclaimed asphalt rejuvenated by crumb rubber modified binder. J. Clean. Prod..

[4] Zhang L., Xing C., Gao F., Li T.-s., Tan Y.-q. (2016). Using DSR and MSCR tests to characterize high temperature performance of different rubber modified asphalt. Constr. Build. Mater..

[5] Ji Z., Wang Z., Feng L., He P., Li S. (2024). Chemical and Rheological Evaluation of the Ageing Behaviour of High-Content Crumb Rubber Asphalt Binder. Polymers.

[6] Yang W., Yi S., Dai T., Li T., Gan Y. (2024). Experimental Research on Anti-UV Aging Performance of Tire Pyrolysis Carbon Black (TPCB) Modified Asphalt. J. China Foreign Highw..

[7] Chen J., Yan K., You L. (2020). Rheological and Spectroscopic Properties of Ethylene Vinyl Acetate–Modified Rubberized Asphalt. J. Mater. Civ. Eng..

[8] Ji W., He D., Wu D., Kargar Razi M. (2021). Research on the Rheological Properties of the Plant Oil Pitch. Int. J. Chem. Eng..

[9] Li S., Chang J., Li Q., Zhou Y., Wang W. (2020). Relationship between chemical and physical or rheological properties of asphalt binders during aging. Pet. Sci. Technol..

[10] Ren S., Liu X., van Aggelen M., Lin P., Erkens S. (2024). Do different chemical and rheological properties act as effective and critical indicators for efficiency evaluation of rejuvenated bitumen?. Constr. Build. Mater..

[11] Li M., Gao Z., He Z., Ma J., Zhao W., Dang S., Wei C. (2024). Effects of Silicone Rubber on Rheological Properties and Aging Characteristics of Asphalt Binder. Polymers.

[12] Chen Z., Wang T., Pei J., Amirkhanian S., Xiao F., Ye Q., Fan Z. (2019). Low temperature and fatigue characteristics of treated crumb rubber modified asphalt after a long term aging procedure. J. Clean. Prod..

[13] Cong P., Xun P., Xing M., Chen S. (2013). Investigation of asphalt binder containing various crumb rubbers and asphalts. Constr. Build. Mater..

[14] Chang M., Zhang Y., Pei J., Zhang J., Wang M., Ha F. (2020). Low-Temperature Rheological Properties and Microscopic Characterization of Asphalt Rubbers Containing Heterogeneous Crumb Rubbers. Materials.

[15] Geng J., Chen M., Xia C., Liao X., Chen Z., Chen H., Niu Y. (2022). Quantitative determination for effective rubber content in aged modified asphalt binder. J. Clean. Prod..

[16] Ma J., Sun G., Sun D., Zhang Y., Cannone Falchetto A., Lu T., Hu M., Yuan Y. (2020). Rubber asphalt modified with waste cooking oil residue: Optimized preparation, rheological property, storage stability and aging characteristic. Constr. Build. Mater..

[17] Zhou T., Zhou J., Li Q., Li B. (2020). Aging Properties and Mechanism of Microwave-Activated Crumb Rubber Modified Asphalt Binder. Front. Mater..

[18] Wang G., Wang X., Lv S., Qin L., Peng X. (2020). Laboratory Investigation of Rubberized Asphalt Using High-Content Rubber Powder. Materials.

[19] Kim H.H., Mazumder M., Torres A., Lee S.-J., Lee M.-S. (2017). Characterization of CRM Binders with Wax Additives Using an Atomic Force Microscopy (AFM) and an Optical Microscopy. Adv. Civ. Eng. Mater..

[20] Lyu L., Fini E.H., Pei J., Poulikakos L.D. (2024). Aging evolution and sustainability implications of crumb rubberized asphalt binder: A state-of-the-art. J. Clean. Prod..

[21] Song L., Xie X., Tu P., Fan J., Gao J. (2023). Study on Aging Mechanism and High-Temperature Rheological Properties of Low-Grade Hard Asphalt. Materials.

[22] Wang H., You Z., Mills-Beale J., Hao P. (2012). Laboratory evaluation on high temperature viscosity and low temperature stiffness of asphalt binder with high percent scrap tire rubber. Constr. Build. Mater..

[23] He Z., Xie T., Yu H., Ge J., Dai W. (2023). Evaluation of quantum dot composite graphene/Titanium oxide enhanced UV aging resistance modified asphalt. Constr. Build. Mater..

[24] Guo M., Yin X., Liang M., Du X. (2023). Study on effect of thermal, oxidative and ultraviolet coupled aging on rheological properties of asphalt binder and their contribution rates. Int. J. Pavement Eng..

[25] Lyu L., Pei J., Hu D., Fini E.H. (2021). Durability of rubberized asphalt binders containing waste cooking oil under thermal and ultraviolet aging. Constr. Build. Mater..

[26] Hu J., Wu S., Liu Q., García Hernández M.I., Zeng W., Xie W. (2017). Study of Antiultraviolet Asphalt Modifiers and Their Antiageing Effects. Adv. Mater. Sci. Eng..

[27] Xiang Y., Xie Y., Long G., Zeng L. (2019). Ultraviolet irradiation of crumb rubber on mechanical performance and mechanism of rubberised asphalt. Road Mater. Pavement Des..

[28] Abouelsaad A., White G. (2022). The Combined Effect of Ultraviolet Irradiation and Temperature on Hot Mix Asphalt Mixture Aging. Sustainability.

[29] Xu Y., Niu K., Zhu H., Chen R., Ou L. (2023). Evaluating the Effects of Polyphosphoric Acid (PPA) on the Anti-Ultraviolet Aging Properties of SBR-Modified Asphalt. Materials.

[30] Wu Y.T. (2017). Low-temperature rheological behavior of ultraviolet irradiation aged matrix asphalt and rubber asphalt binders. Constr. Build. Mater..

[31] Xu S.F., Ren X.Y., Wu H.L., Liu H.Z., Xu M., Zhu Z.X., Ling M. (2024). Effect of Ultraviolet Aging on Fundamental Properties of Polymer and Crumb Rubber Modified Asphalt and Asphalt Mixtures. J. Mater. Civ. Eng..

[32] Ju Z., Ge D., Xue Y., Duan D., Lv S., Cao S. (2024). Investigation of the influence of the variable-intensity ultraviolet aging on asphalt properties. Constr. Build. Mater..

[33] Zadshir M., Ploger D., Yu X., Sangiorgi C., Yin H. (2020). Chemical, thermophysical, rheological, and microscopic characterisation of rubber modified asphalt binder exposed to UV radiation. Road Mater. Pavement Des..

[34] Jamal M., Lanotte M., Giustozzi F. (2022). Exposure of crumb rubber modified bitumen to UV radiation: A waste-based sunscreen for roads. J. Clean. Prod..

[35] Jamal M., Giustozzi F. (2022). Enhancing the asphalt binder’s performance against oxidative ageing and solar radiations by incorporating rubber from waste tyres. Constr. Build. Mater..

[36] Gao M., Fan C., Chen X., Li M., Abdul Khalil H.P.S. (2022). Study on Ultraviolet Aging Performance of Composite Modified Asphalt Based on Rheological Properties and Molecular Dynamics Simulation. Adv. Mater. Sci. Eng..

[37] Zhang D., Huang Z., Yuan G., Zheng Y., Qian G., You Z., Zhang H. (2022). Research on the anti-aging mechanism of SBS-modified asphalt compounded with multidimensional nanomaterials based on atomic force microscopy. Constr. Build. Mater..

[38] Borinelli J.B., Enfrin M., Blom J., Giustozzi F., Vuye C., Hernando D. (2024). Investigating thermal and UV ageing effects on crumb rubber modified bitumen enhanced with emission reduction agents and carbon black. Constr. Build. Mater..

[39] Ju Z., Ge D., Zhang H., Lv S., Xue Y. (2024). Rheological behavior and microscopic characteristic of electromagnetic thermal activated crumb rubber and SBS modified asphalt. Constr. Build. Mater..

[40] (2006). Standard Method of Test for the Penetration of Bituminous Materials.

[41] (2013). Standard Method of Test for Ductility of Asphalt Materials.

[42] (2016). Standard Test Method for Softening Point of Bitumen (Ring-and-Ball Apparatus).

[43] (2019). Standard Method of Test for Specific Gravity of Semi-Solid Asphalt Materials (Pycnometer Method).

[44] (2019). Standard Method of Test for Flash Point by Cleveland Open Cup.

[45] Ju Z., Ge D., Wu Z., Xue Y., Lv S., Li Y., Fan X. (2022). The performance evaluation of high content bio-asphalt modified with polyphosphoric acid. Constr. Build. Mater..

[46] (2019). Standard Method of Test for Effect of Heat and Air on a Moving Film of Asphalt Binder (Rolling Thin-Film Oven Test).

[47] (2019). Standard Method of Test for Determining the Rheological Properties of Asphalt Binder Using a Dynamic Shear Rheometer (DSR).

[48] Yang X., You Z., Dai Q., Mills-Beale J. (2014). Mechanical performance of asphalt mixtures modified by bio-oils derived from waste wood resources. Constr. Build. Mater..

[49] (2014). Standard Method of Test for Multiple Stress Creep Recovery (MSCR) Test of Asphalt Binder Using a Dynamic Shear Rheometer.

[50] (2014). Standard Method of Test for Estimating the Fatigue Life of Asphalt Binders Using the Linear Amplitude Sweep (LAS) Test.

[51] (2019). Standard Method of Test for Determining the Flexural Creep Stiffness of Asphalt Binder Using the Bending Beam Rheometer (BBR).

[52] Hou X., Lv S., Chen Z., Xiao F. (2018). Applications of Fourier transform infrared spectroscopy technologies on asphalt materials. Measurement.

[53] Fan T., Han S., Si C. (2025). Fatigue performance of calcium sulfate whisker-modified asphalt under multi-modal aging conditions. J. Clean. Prod..

[54] Islam M.R., Salomon D., Wasiuddin N.M. (2024). Investigation of oxidative aging of field-extracted asphalt binders at various conditions using carbonyl index. Constr. Build. Mater..

[55] Yang J., Zhang Z., Xue J., Lei J.a., Liu Y., Wang Y., Fang Y. (2023). Variation and mechanism of asphalt-aggregate interface features under ultraviolet aging based on meso- and micro-observations. Constr. Build. Mater..

[56] Jamal M., Martinez-Arguelles G., Giustozzi F. (2021). Effect of waste tyre rubber size on physical, rheological and UV resistance of high-content rubber-modified bitumen. Constr. Build. Mater..

[57] Liu Y.-R., Tang X., Zeng Q., Lai J.-P. (2024). Impacts of ultraviolet absorption by zinc oxide nanoparticle modifiers on asphalt aging. Sci. Rep..

[58] Li H., Yu J., Wu S., Pang L., Li Y., Wu Y. (2018). Property of Anti-Ultraviolet Aging of LDHs Modified Asphalt. J. Wuhan Univ. Technol.-Mater. Sci. Ed..

[59] Ma Y., Wang S., Zhang M., Jiang X., Polaczyk P., Huang B. (2023). Weather aging effects on modified asphalt with rubber-polyethylene composites. Sci. Total Environ..

[60] Lee S.-J., Hu J., Kim H., Amirkhanian S.N., Jeong K.-D. (2011). Aging analysis of rubberized asphalt binders and mixes using gel permeation chromatography. Constr. Build. Mater..

[61] Lin M., Shuai J., Li P., Kang X., Lei Y. (2022). Analysis of rheological properties and micro-mechanism of aged and reclaimed asphalt based on multi-scales. Constr. Build. Mater..

[62] Bendjaouahdou C., Bensaad S. (2018). Aging studies of a polypropylene and natural rubber blend. Int. J. Ind. Chem..

